# Impacts of parental technoference on parent-child relationships and child health and developmental outcomes: a scoping review protocol

**DOI:** 10.1186/s13643-022-01918-3

**Published:** 2022-03-17

**Authors:** Lyndsay Jerusha Mackay, Jelena Komanchuk, K. Alix Hayden, Nicole Letourneau

**Affiliations:** 1 Faculty of Nursing, University of Calgary, 2500 University Drive NW, Calgary, Alberta T2N 1N4 Canada; 2 Libraries & Cultural Resources, University of Calgary, 2500 University Drive NW, Calgary, Alberta T2N 1N4 Canada; 3 Faculty of Nursing, Cumming School of Medicine, Pediatrics, Psychiatry & Community Health Sciences, University of Calgary, 2500 University Drive NW, Calgary, Alberta T2N 1N4 Canada

**Keywords:** Technoference, Parent-child relationships, Parent-child interactions, Bonding: Attachment, Child health and development, Phubbing, Parent media distraction, Technology use, Smartphone use

## Abstract

**Background:**

With increases in the use of technological devices worldwide, parental technoference is a potential threat to the quality of parent-child relationships and children’s health and development. Parental technoference refers to disrupted interactions between a parent and child due to a parent’s use of a technological device. The aims of this scoping review are to map, describe, and summarize the existing evidence from published research studies on the impacts of parental technoference on parent-child relationships and children’s health and development and to identify the limitations in the studies and gaps in the literature.

**Methods:**

This scoping review will be conducted in accordance with the Joanna Briggs Institute (JBI) methodology. A search for relevant research studies will be undertaken in APA PsycInfo, MEDLINE, Central, Cochrane Database for Systematic Reviews, JBI EBP, and Embase (OVID). CINAHL (Ebsco) and Scopus will also be searched. Grey and popular literature will be excluded. This review will include primary research studies and review papers published in English with no time limit that identify the impacts of technoference on parent-child relationships and child health and developmental outcomes. Parent participants include primary caregivers, either biological, adopted, or foster parents, of children under the age of 18 who engage in technoference. Two reviewers will independently screen the titles, abstracts, and full texts of studies according to the inclusion and exclusion criteria. Disagreements will be resolved through discussion with a third researcher. Data will be extracted into a data charting table including author(s), year of publication, country, research aim, methodology/design, population and sample size, variables/concepts, and corresponding measures and main results. Data will be presented in tables and figures accompanied by a narrative summary.

**Discussion:**

The goal of this scoping review is to present an overview of the evidence on the impacts of parental technoference on parent-child relationships and child and health developmental outcomes, highlighting the current risk of children of today. It will identify gaps in the literature, inform future research, advise recommendations for parents on technological device use, and possibly guide the development of interventions aimed at addressing parental technoference.

**Trial registration:**

Open Science Framework 10.17605/OSF.IO/QNTS5

**Supplementary Information:**

The online version contains supplementary material available at 10.1186/s13643-022-01918-3.

## Background

Mobile devices are becoming ubiquitous worldwide. In 2018, 88% of Canadians owned a mobile device, and 87% had internet access [1]. Similarly, in 2019, 96% of Americans owned a cell, of which 81% were smartphones, up from 35% in 2011 [2]. Ninety percent of Americans frequently carry their cell phones, and 45% rarely turn them off [3]. In 2019, 86% of the population in Europe subscribed to mobile services, and 76% of the connections were via a smartphone [4]. In 2018, the overall mobile use among countries in South Asia was 33%, with India at 55%, Bangladesh at 22%, and Pakistan at 22% [5]. In Southeast Asia in 2018, 42% of the population had smartphones, and 28% owned another type of phone [5]. Forty-five percent of the population in sub-Saharan Africa subscribed to mobile services at the end of 2019 [6], and 70% of the population in Latin America were cell phone subscribers by the end of 2019 [7]. Globally, mobile device use is soaring, which has resulted in an “always-on” reality that has caused disruptions to social norms about the appropriateness of diverting one’s attention from a relational interaction to a digital encounter [3].

Today’s parents spend significant amounts of time on technological devices. In one study, mothers of infants (*N* = 114) spent an average of 3 h per day on their smartphones; 16% of mothers reported spending 5–15 h on their smartphones per day, and 6.7% described being addicted to their smartphones [8]. The majority of mothers (75%) used their smartphones primarily for entertainment and social-networking purposes [8]. Another study among mothers (*N* = 553) and their children found that mothers spent an average of 4.33 h per day on a technological device [9]. High screen time in mothers was associated with conduct problems, symptoms of hyperactivity/inattention, and emotional problems among their children [9]. Findings from an observational study of parents’ (*N* = 50) use of mobile devices while caring for their children at a playground revealed that 76% of parents used their mobile device for up to 17.5 min of the 20 min observation period [10]. Parents are spending high amounts of time each day on a technological device, which leads to the question: does parents’ high technological device use interfere in regular parenting behaviors and distract them from engaging in optimal parent-child interactions, negatively impacting child health and development?

Parental technoference is defined as regular interruptions to real-time face-to-face communications, interactions, or time spent together between family members because of parental use of technology [11, 12]. This includes periods of time when a parent checks a technological device during family interactions that creates feelings of intrusion [11]. Technoference has become common among families with children [13], and researchers are beginning to identify that extended parental time on technological devices can have negative effects on parent-child relationships and children’s health and developmental outcomes [14].

Definitions of parent-child relationships typically refer to the qualities of parents’ interactions with their children [15, 16], as well as bonding [17] and attachment [18]. Optimal parent-child interactions are observed when a child verbalizes, gestures, or cries and the parent responds appropriately with verbalization, eye contact, or physical touch [15]. Parent-child interactions involve how parents and children affect the behavior of one another by a process of bi-directional influences and social interaction [16]. Through this process, the child and parent learn to adapt and modify their behaviors in response to one another [16]. Parents’ ability to respond to their children’s cues (e.g., crying) and bids for attention (e.g., reaching for parent) in a supportive and responsive manner results in optimal parent-child interactions [19]. Supportive parent-child interactions are the single most important factor known to promote optimal child development, including positive emotional, behavioral, psychological, and social development [20], and have been shown to support children’s health [21, 22]. Parental technoference can negatively impact parents’ interactions with their children of all ages because it reduces parental attention, responsiveness, and warmth displayed to their children [23, 24]. When parental technoference was observed in playgrounds and eateries in the USA and Israel, parents’ interactions with their children were suboptimal. Such parents regularly ignored their children’s bids for attention and were inattentive to their children’s emotional and safety needs due to focusing their attention on a mobile device [25].

Bonding refers to a specific relationship between two people that endures over time [17]. In reference to parent-child bonding, bonding refers to the emotional tie a mother or father forms with their newborn infant as they become dedicated to caring for their infant and demonstrate behaviors of care, concern, and affection towards their infant (e.g., kissing, cuddling, and prolonged gaze) [17, 26]. Most often, this emotional tie is from the mother towards her infant, not the infant to the mother as is referred to in attachment [27]. Attachment refers to children’s behavior of seeking or maintaining close proximity to a caregiver that they identify as being safe, able to meet their needs, and offering protection [18]. Children’s attachment behaviors to their caregivers are most evident when children feel sick, frightened, or fatigued, as demonstrated by their internal motivation to seek comfort from their caregiver [18]. Most often, the caregiver is the children’s mother-figure, but in her absence, children can demonstrate attachment towards someone they feel comfortable with or know well [18].

Childhood is characterized by a sensitive period of brain and biological development, leaving children influenced by the environment in which they live, which can improve or damage their development and health [28, 29]. Children’s development includes several interdependent domains, including gross motor (e.g., sitting, standing, walking, running), fine motor (e.g., eating, writing), language (e.g., speaking, gestures), social (e.g., relationships with others, responding to others’ feelings), and cognitive (e.g., learning, understanding, problem-solving, reasoning, remembering) [30, 31]. Children’s health includes biological health (e.g., cardiac, respiratory, endocrine, muscular-skeletal, and gastroenteric domains) and psychological health (e.g., mental, emotional, and behavioral domains). Parental technoference can interfere with children’s health and development, specifically predicting children’s externalizing (e.g., hyperactivity, aggression) and internalizing (e.g., anxiety, depression) behavioral problems [24, 32].

A preliminary search of MEDLINE, Cochrane Database for Systematic Reviews, and Joanna Briggs Institute (JBI) Evidence Synthesis was conducted. One systematic review was retrieved on the impact of parental smartphone use on parent-child interactions [23]. This systematic review excluded children’s outcomes other than parent-child interactions. One review on the impacts of parental distraction with phones on parenting and child outcomes was found [13]. However, this review did not follow a systematic review method, include impacts on parent-child relationships, nor was a comprehensive search of the literature conducted; all of which we will perform for this scoping review. Finally, a systematic review was retrieved on parents’ use of mobile computing devices on caregiving and social and emotional development of children [33]. This review included articles that reported parents’ use of mobile devices rather than technoference, which considers when parents become distracted from an interaction due to the use of technology. Given that parents may utilize technological devices when they are apart from their children (e.g., while their child is asleep or at school), we will include articles that identify the outcomes in relation to parental technoference and exclude articles that only report parental use of a technological device.

The aim of this scoping review is to map, describe, and summarize peer-reviewed evidence on the impacts of parental technoference on parent-child relationships and child health and developmental outcomes. This scoping review will identify the current state of the literature and discover gaps in the literature, which will help inform future research that provides a comprehensive understanding and guides the development of interventions and strategies aimed at buffering children from the harmful impacts of parental technoference.

## Methods

### Review questions

The primary research question for this review is: What research evidence is available on the impacts of parental technoference on parent-child relationships and children’s health and developmental outcomes? Sub-questions for this review include: (1) What are the gaps in relevant literature? (2) How is parental technoference defined in the literature? (3) How is parental technoference measured in the literature? and (4) What research designs and methodologies are used to research parental technoference?

### Protocol design

The purpose of this review is not to identify the effectiveness of a treatment or practice as is most often the case with systematic reviews [34, 35]. Rather, the objective of this review is to identify and map types of the available evidence, examine how the research was conducted, identify the knowledge gaps, and synthesize and summarize the available evidence on the impacts of parental technoference on parent-child relationships and child health and developmental outcomes; thus, a scoping review methodology was chosen [34, 36]. The proposed scoping review will be conducted in accordance with the JBI methodology for scoping reviews [37] and reported in accordance with the Preferred Reporting Items for Systematic Reviews (PRISMA) Statement for Scoping Reviews (PRISMA-ScR) (see Additional file 1) [38]. It has been registered with the Open Science Framework (registration number: 10.17605/OSF.IO/QNTS5). Any deviations from this protocol will be reported in the final manuscript.

### Eligibility criteria

The participant, concept, and context (PCC) framework for scoping reviews will be used to inform the eligibility of research articles and guide the review process [37] (see Additional file 2).

#### Participants

This review will consider studies that include parents who engage in technoference and their children. Parents must be the primary caregivers, including married, unmarried, divorced, separated, biological, adoptive, and foster parents. Children do not have to reside in the same home as their parental caregivers. Parents of all ages will be included, such as older parents and teenage parents. Children must be under the age of 18 and can be identified as healthy or with a disability/disease of any kind.

#### Concept

This review will consider studies that explore technoference among parents. Technoference is defined as parents’ use of technological devices that interferes with or interrupts everyday normal family relations and interactions, including but not limited to face-to-face conversations, mealtimes, and leisurely time together. Technological devices include any technological device that distracts parental attention from their child/children, such as a cellphone, smartphone, or tablet, excluding watching a television.

Parent-child relationships reflect the nature of interactions between parents and their child/children. This can include but is not limited to parent-child interaction quality (e.g., sensitivity and responsiveness), attachment, and bonding.

Child health involves biological and psychological health. Biological health includes, but is not limited to, cardiac, respiratory, muscular-skeletal, endocrine, and gastroenteric domains. Psychological health includes, but is not limited to, mental, emotional, and behavioral domains.

Child development includes, but is not limited to, gross motor, fine motor, language, social, and cognitive domains.

#### Context

Technoference can occur in any context with no limits on cultural factors, geographic location, specific setting, or racial and gender-based interests.

#### Types of sources

This scoping review will consider both experimental and quasi-experimental study designs including randomized controlled trials, non-randomized controlled trials, before and after studies, and interrupted time-series studies. In addition, analytical observational studies including prospective and retrospective cohort studies, case-control studies, and analytical cross-sectional studies will be considered for inclusion. This review will also consider descriptive observational study designs including case series, individual case reports, and descriptive cross-sectional studies for inclusion, along with systematic reviews, meta-analysis, and scoping reviews. Qualitative studies will also be considered including, but not limited to, designs such as phenomenology, grounded theory, ethnography, qualitative description, action research, and feminist research. In addition, conference abstracts, reviews, and dissertations that meet the inclusion criteria will also be considered. Grey literature, popular literature, letters, editorials, and opinions will be excluded. Studies published in English will be included with no specific date limit.

### Search strategy

The search strategy will aim to locate published studies. An initial discovery search of APA PsycInfo will be undertaken to identify articles on the topic. The text words contained in the titles and abstracts of relevant articles, and the index terms used to describe the articles, will be used to develop a full search strategy focusing on three main concepts: parents, children, and technoference. Outcome terms related to child development and health will not be searched as searching outcomes has been shown to impact retrieval of relevant studies [39]. The search strategy, including all identified keywords and index terms, will be adapted for each included database and/or information source. A draft search strategy for APA PsycInfo is provided in Additional file 3. The reference list of all included sources of evidence will be screened for additional studies.

Ovid databases to be searched include the following: APA PsycInfo, MEDLINE(R) and Epub Ahead of Print, In-Process & Other Non-Indexed Citations and Daily, Cochrane Central Register of Controlled Trials (Central), Cochrane Database for Systematic Reviews, JBI EBP Database, and Embase. CINAHL Plus with Full Text (Ebsco) and Scopus will also be searched.

### Study/source of evidence selection

Following the search, all identified records will be uploaded into Covidence [40] and duplicates removed. Two reviewers (LM and JK) will pilot the eligibility criteria on 50 random titles and abstracts to gain a minimum of 90% inter-rater reliability. Following the calibration exercise, titles and abstracts will then be screened by two independent reviewers (LM and JK) for the assessment against the inclusion criteria for the review. Potentially relevant studies will be retrieved in full text and uploaded to Covidence. The full texts will be assessed in detail against the inclusion criteria by two independent reviewers (LM and JK). Reasons for the exclusion of sources of evidence in the full text that do not meet the inclusion criteria will be recorded and reported in the scoping review. Any disagreements that arise between the reviewers at each stage of the selection process will be resolved through discussion, or with an additional reviewer (NL). The results of the search and the study inclusion process will be reported in full in the final scoping review through a PRISMA-ScR flow diagram [38].

### Data extraction

Data will be extracted from papers using a modified version of the JBI data extraction instrument [37] (see Additional file 4). Extracted data will include specific details about the authors, year of publication, country, participants, concept, context, study methods, and key findings relevant to the review questions. The data extraction form will be piloted by two reviewers on five research studies and cross-checked by a third reviewer. Necessary adjustments will be made to the data extraction form. If the data extraction instrument is modified during the process of extracting data, this will be detailed in the scoping review. Data will be independently extracted by one researcher, and validity of the extracted data will be ensured by a second researcher cross-checking the extracted data. If appropriate, the authors of the papers will be contacted to request for missing or additional data.

### Critical assessment of evidence

Critical appraisal is not mandatory within the JBI methodology for scoping reviews; however, such appraisal is useful to report the risk of bias in scoping reviews [37]. The Newcastle-Ottawa Scale (NOS) will be used to assess the quality of non-randomized studies [41], the Consolidated Criteria for Reporting Qualitative Studies (COREQ) will be used to assess the quality of qualitative studies [42], and the Consolidated Standards of Reporting Trials (CONSORT) will be used to assess the quality of randomized controlled trials [43]. Assessment of each study will be assessed by two independent researchers (LM and JK). If disagreements arise, a third researcher will be utilized to come to a consensus (NL). Critical assessment of evidence will be included on the data extraction form, presented in tables and figures, and addressed in the narrative summary.

### Data analysis and presentation

Data will be descriptively mapped with simple frequency counts of concepts, populations, and characteristics if such data are available. Tables and figures will present the distribution of articles by period of publication, county of origin, type of outcome (e.g., parent-child interactions, child health, and/or child development), technoference measurement tool, age of children (e.g., early childhood, childhood, and adolescence), and research method. A narrative summary of the study findings will accompany the tables and figures that describe the impacts of technoference on parent-child interactions and child health and developmental outcomes from the literature.

## Discussion

The concept of technoference is a relatively new term. It has recently emerged as researchers and scholars begin to recognize the negative effects parental technoference can have on parent-child relationships and children’s health and development. This scoping review will provide a descriptive overview of the available evidence on the impacts of parental technoference on parent-child relationships and children’s health and development.

This scoping review will result in tables and figures that map the year, authors, ages of children, setting, study design, and outcomes measures used in relevant studies, along with a supporting narrative summary. It will also identify the gaps in the existing literature, including areas that require further investigation. The various assessment tools used to measure parental technoference will be identified along with definitions of parental technoference. The above will help inform future research aimed at increasing understanding and knowledge of the impacts of parental technoference on parent-child relationships and children’s health and developmental outcomes and aid in the development of programs and interventions that buffer children from the harmful effects of parental technoference and reduce parental technoference.

It is hoped that by publishing this protocol and the subsequent scoping review results, practitioners, researchers, scholars, policymakers, and parents of today will become aware of and begin discussing the implications that parental technoference has for children. The results of this scoping review will be disseminated through peer-reviewed publications and presented at international, national, and local conferences. The findings will be used in discussion with policymakers and practitioners to inform policy and practice on recommendations made to parents regarding their technological device use when in the presence of their children.

Although rigor has been applied to this scoping review protocol, potential limitations may occur. There may be studies published in languages other than English that are relevant and not included. Evidence published in grey literature, such as government reports, may not be captured. Protocol amendments will be documented and captured in the final publication of the scoping review results.

## Supplementary Information

**Additional file 1.** Preferred Reporting Items for Systematic reviews and Meta-Analyses extension for Scoping Reviews (PRISMA-ScR) Checklist.

**Additional file 2.** PCC framework defining eligibility.

**Additional file 3.** Draft search strategy for APA PsycInfo.

**Additional file 4.** Data extraction table.

## Data Availability

Not applicable
