# Supplementary material for: Impacts of parental technoference on parent-child relationships and child health and developmental outcomes: a scoping review protocol
Source: Syst Rev. 2022 Mar 17;11:45. doi: 10.1186/s13643-022-01918-3 (PMC8932188; doi:10.1186/s13643-022-01918-3)
Supplement: Supplementary file 2 — Additional file 2. PCC framework defining eligibility. [file 13643_2022_1918_MOESM2_ESM.docx]

**Additional file 2: PCC framework defining eligibility**

| Population | Parents:   - Who engage in technoference - Who are the primary caregiver of a child, including;  1. Married 2. Unmarried 3. Divorced 4. Separated 5. Biological caregiver 6. Adoptive caregiver 7. Foster parent   Children:   - Must be under the age of 18 - Do not have to reside in the same home as their parent - Identified as healthy or with a disability/disease of any kind |
| --- | --- |
| Concept | Technoference:   - Parental use of technological devices that interfere or interrupt with normal family relations and interactions with their children - Interactions include but are not limited to; face-to-face conversations, mealtimes and leisurely time together - Technological devices include but are not limited to; cell phone, smart phone, tablet (e.g., iPad) - Excluding television   Parent-Child Relationship:   - Including: Parent-child interactions, attachment and bonding  1. Parent-child interactions: when a child verbalizes, gestures or cries and the parent responds appropriately 2. Attachment: children’s behavior of seeking or maintaining close proximity to a caregiver 3. Bonding: emotional tie a caregiver forms with their child   Child Development:   - Includes; gross motor, fine motor, language, social and cognitive   Child Health:   - Biological health, including; cardiac, respiratory, muscular-skeletal, endocrine and gastroenteric - Psychological health, including; mental, emotional and behavioral |
| Context | Setting:   - Parental technoference can occur in any location, including but not limited to; home environment, playground, educational environment, sport facility and family gatherings.   Cultural Factor:   - Any, no limitations   Geographic Location:   - Any country, no limitations   Racial and gender-based interests:   - Any, no limitations |
