# Supplementary material for: Impacts of parental technoference on parent-child relationships and child health and developmental outcomes: a scoping review protocol
Source: Syst Rev. 2022 Mar 17;11:45. doi: 10.1186/s13643-022-01918-3 (PMC8932188; doi:10.1186/s13643-022-01918-3)
Supplement: Supplementary file 3 — Additional file 3. Draft search strategy for APA PsycInfo. [file 13643_2022_1918_MOESM3_ESM.docx]

**Additional file 3: Draft search strategy for APA PsychInfo**

Search Strategy:

| **#** | **Searches** |
| --- | --- |
| 1 | exp parents/ or exp mothers/ or exp fathers/ or exp stepparents/ |
| 2 | exp Coparenting/ or exp Parenting/ |
| 3 | exp Couples/ or exp Same Sex Couples/ |
| 4 | (maternal or paternal or parental).tw,id. |
| 5 | (mother* or father* or parent* or step-parent* or stepparent* or foster parent* or couple*).tw,id. |
| 6 | (mom or moms or dad or dads).tw,id. |
| 7 | or/1-5 |
| 8 | exp mobile devices/ or exp mobile phones/ or exp tablet computers/ or exp text messaging/ or social media/ |
| 9 | smartphones/ or "smartphone use"/ |
| 10 | ((smartphone* or smart phone* or mobile phone* or cell phone* or phone* or iphone* or technolog* or digital media or social media or texting) adj5 ("use" or using or usage or distract* or interference or interfer* or interruption*)).tw,id. |
| 11 | (instagram or facebook or twitter or snapchat or snap chat or pinterest or tiktok or tik tok).tw,id. |
| 12 | or/8-11 |
| 13 | pediatrics/ |
| 14 | (pediatric* or paediatric*).tw,id. |
| 15 | (infant* or baby or babies or newborn*).tw,id. |
| 16 | (toddler* or preschool* or pre-school* or child*).tw,id. |
| 17 | (boy or boys or girl or girls*).tw,id. |
| 18 | (son or sons or step-son* or stepson* or daughter* or step-daughter* or stepdaughter*).tw,id. |
| 19 | (pubescen* or juvenile* or teen* or tween* or youth* or high school* or adolesc* or pre-pubesc* or prepubesc*).tw,id. |
| 20 | (child* or adolesc* or pediat* or paediat*).jx. |
| 21 | or/13-20 |
| 22 | 7 and 12 and 21 |
| 23 | (technoference or phubbing).tw,id. |
| 24 | 22 or 23 |
