# Supplementary material for: Impacts of parental technoference on parent-child relationships and child health and developmental outcomes: a scoping review protocol
Source: Syst Rev. 2022 Mar 17;11:45. doi: 10.1186/s13643-022-01918-3 (PMC8932188; doi:10.1186/s13643-022-01918-3)
Supplement: Supplementary file 4 — Additional file 4. Data extraction table. [file 13643_2022_1918_MOESM4_ESM.docx]

**Additional file 4: Data extraction table**

Reviewer:

| **Publication Details** | |
| --- | --- |
| Authors |  |
| Year |  |
| Article title |  |
| Journal, volume, issue, page numbers |  |
| **Study design and details** | |
| Aim/purpose |  |
| Research Questions |  |
| Hypothesis(es) |  |
| Setting (country; characteristics of setting) |  |
| Sampling technique (convenience, probability) |  |
| Sample size |  |
| Details of study participants (age/sex of parents and children; characteristics of parents/children) |  |
| Study design |  |
| Variables |  |
| Method of data collection (questionnaires; interviews; observation) |  |
| Measurement tool (survey; questionnaire) |  |
| Method of data analysis |  |
| **Outcomes/Details of Results** | |
| Main/key findings |  |
| Technoference specific |  |
| Parent-child relationships |  |
| Child health specific |  |
| Child development specific |  |
| **Study Appraisal (New-Castle, CONSORT or COREQ)** | |
|  | |

Date of data extraction:
